# School-based vaccination programmes: a systematic review of the evidence on organisation and delivery in high income countries

**DOI:** 10.1186/s12889-017-4168-0

**Published:** 2017-03-14

**Authors:** Sarah Perman, Simon Turner, Angus I. G. Ramsay, Abigail Baim-Lance, Martin Utley, Naomi J. Fulop

**Affiliations:** 1 Department of Applied Health Research, UCL, 1-19 Torrington Place, London, WC1E 7HB UK; 2 Clinical Operational Research Unit, UCL, 4 Taviton Street, London, WC1H 0BT UK; 3 Present address: Redbridge Clinical Commissioning Group, Becketts House, 2-14 Ilford Hill, Ilford, IG1 2QX UK

**Keywords:** Schools, Vaccination, Mass vaccination, Implementation, Organisational change

## Abstract

**Background:**

Many countries have recently expanded their childhood immunisation programmes. Schools are an increasingly attractive setting for delivery of these new immunisations because of their ability to reach large numbers of children in a short period of time. However, there are organisational challenges to delivery of large-scale vaccination programmes in schools. Understanding the facilitators and barriers is important for improving the delivery of future school-based vaccination programmes.

**Methods:**

We undertook a systematic review of evidence on school-based vaccination programmes in order to understand the influence of organisational factors on the delivery of programmes. Our eligibility criteria were studies that (1) focused on childhood or adolescent vaccination programmes delivered in schools; (2) considered organisational factors that influenced the preparation or delivery of programmes; (3) were conducted in a developed or high-income country; and (4) had been peer reviewed. We searched for articles published in English between 2000 and 2015 using MEDLINE and HMIC electronic databases. Additional studies were identified by searching the Cochrane Library and bibliographies. We extracted data from the studies, assessed quality and the risk of bias, and categorised findings using a thematic framework of eight organisational factors.

**Results:**

We found that most of the recent published literature is from the United States and is concerned with the delivery of pandemic or seasonal flu vaccination programmes at a regional (state) or local level. We found that the literature is largely descriptive and not informed by the use of theory. Despite this, we identified common factors that influence the implementation of programmes. These factors included programme leadership and governance, organisational models and institutional relationships, workforce capacity and roles particularly concerning the school nurse, communication with parents and students, including methods for obtaining consent, and clinic organisation and delivery.

**Conclusions:**

This is the first time that information has been brought together on the organisational factors influencing the delivery of vaccination programmes in school-based settings. An understanding of these factors, underpinned by robust theory-informed research, may help policy-makers and managers design and deliver better programmes. We identified several gaps in the research literature to propose a future research agenda, informed by theories of implementation and organisational change.

**Electronic supplementary material:**

The online version of this article (doi:10.1186/s12889-017-4168-0) contains supplementary material, which is available to authorized users.

## Background

Schools have become an increasingly important setting for delivery of immunisation programmes in high income countries. Beginning in some countries with polio vaccination in the 1950s, generations of children have received vaccinations through school-based programmes. Many countries have recently expanded their childhood immunisation programmes to incorporate new vaccinations such as annual intranasal influenza vaccine for healthy children and Human Papillomavirus (HPV) vaccination for teenage girls [1]. Schools are an attractive venue for providing these vaccines because of their ability to reach large numbers of children in a short period of time.

Evidence from a wide range of studies shows that school-based vaccination is effective in achieving high uptake and completion rates [2–5]. This evidence comes from different types of school-based programmes: routine and booster immunisation programmes, catch up programmes for unvaccinated and partially vaccinated young people, and vaccination in response to an outbreak of disease. Research also shows that school-based vaccination is successful in reducing the burden of disease in the wider community as well as in the vaccinated population [4, 6, 7]. Studies have found school-based vaccination to be acceptable to education staff, health professionals, parents and students even when programmes involve new vaccines and parental concerns figure strongly [8, 9].

There are, however, considerable political, organisational and logistical challenges to delivery of such large scale programmes in schools. Challenges include which organisational and funding models should be selected, questions about vaccine supply and distribution, issues around staff capacity and workload, as well as how to inform parents, obtain consent, and minimise anxiety and distress to students. The exact nature of these challenges and approaches for successfully overcoming them are not currently well understood.

With the advent of further new vaccines on the horizon, it is likely that mass vaccination programmes will be a more frequent event in the school timetable [10]. Understanding the processes that influence school-based vaccination programmes is important information for shaping strategy and policy for future programmes. Our review aims to identify the factors that influence successful delivery in order to support more effective programme design and implementation. We aimed to identify the contextual and organisational influences, enablers and barriers which impact on the delivery of programmes in school-based settings.

## Methods

We undertook a systematic review using a narrative synthesis approach. Narrative synthesis is an established method used in systematic reviews to review and integrate diverse forms of evidence [11, 12]. Evidence for organisational influences on school-based vaccination can be found in heterogeneous studies, including process evaluations which vary in the degree to which formal research methods are used. This approach suited our research question because it enabled us to look for, include and synthesise evidence from different types of studies.

Our first step was to conduct a scoping review of the literature to enable us to trial search terms for our full review, and to identify emerging themes for coding our results. Our scoping review generated eight broad organisational factors that researchers across different studies and from different countries identified as significant influences on the delivery of school-based programmes. We used these eight factors to develop a thematic framework consisting of inductively-constructed codes which we would later use to group our findings from the studies we included in our full review (Table 1).

**Table 1:** Coding framework for thematic analysis based on organisational influences on the implementation of school—based programmes

Theme code	Organisational factors	Theme explanation
A	National and regional policy	Directly related policy, e.g. the aims/target group for the vaccination programme Indirectly related policy, e.g. education policy, health service policy
B	Programme management and leadership	Leadership and management of the programme at local, area and national levels
C	Organisational models and institutional relationships	Models of programme organisation and inter-organisational communication and collaboration
D	Infrastructure	Facilities and systems for the programme, e.g. systems for vaccine distribution and supply, data systems for vaccination records
E	Workforce: capacity and activity	Staff capacity, workload, skill, experience and roles
F	Programme financing	Resourcing, billing and reimbursement, sustainability
G	Communication with parents and students	Practical issues, e.g. distribution of consent forms and obtaining consent Conceptual issues, e.g. tailoring messages, involving children and adolescents
H	Clinic organisation and delivery	Logistics on the day, physical configuration of clinics

For our full review, we conducted searches on two electronic databases: MEDLINE and Health Management Information Consortium (HMIC — a bibliographic database of health management and policy). For our searches we used the National Institute for Health and Care Excellence (NICE) search platform (https://www.evidence.nhs.uk). We limited our search to studies in English and published between 2000 and 2015. The year 2000 was our cut-off due to limited capacity in our team. Our last search was conducted on 30 August 2015. One of our searches is reproduced in full in an appendix [see Additional file 1]. We searched the Cochrane library by topic and review. In addition, we hand searched public health journals, looked for studies cited in systematic reviews, and contacted one study author to obtain an additional article.

We screened studies by title and abstract, and retrieved full text articles. Studies were included if they met the following criteria: (1) focused on childhood or adolescent vaccination programmes delivered in schools; (2) considered organisational factors that influenced the preparation or delivery of programmes; (3) were conducted in a developed or high-income country; and (4) had been peer reviewed. We restricted our study to high income countries because we were interested in comparing results for countries with similar vaccination schedules and with more developed health and education systems. As long as the above criteria were met, studies of any vaccination programme were included: i.e. covering routine immunisations, catch up programmes for unvaccinated or partially vaccinated children, and vaccinations in response to a disease outbreak. We excluded studies that examined attitudes to and beliefs about vaccination, focused on impact with no analysis of process, or involved the use of schools for immunisation of the local community rather than school students.

We coded the included studies by study type, country and vaccine programme (Tables 2 and 3). For study type, we classified studies into five categories (I to V) based on main methods reported. We appraised the quality of studies in three of our categories (I, II and III), including an assessment of the risk of bias, using checklists that we adapted from the UK’s Critical Appraisal Skills Programme. These checklists are shown in an appendix [see Additional file 2]. We did not assess the quality of descriptive studies and expert opinion pieces (i.e. categories IV and V) as there was insufficient detail within these studies on methods used. We appraised the quality of papers in order to describe that quality and to inform our synthesis of findings rather than to include or exclude [13]. In our analysis, we gave papers of lower quality less weight than those that were of higher quality.

**Table 2:** Included studies by study type, coded by main methods used

Study type	Examples of methods used	Number of studies
I Reviews/synthesis of studies	Systematic review, narrative review	3
II Quantitative	Cohort analysis, cross-sectional survey, economic evaluation	7
III Qualitative	Focus groups, semi-structured interviews, observations	15
IV Descriptive	Descriptions of programme experiences, local evaluations	16
V Non-research	Expert opinion/conference paper	3
	TOTAL	44

**Table 3:** Included studies by country and vaccine disease type

Country and vaccine type	Number of studies	Examples
Australia, HPV	2	Bernard 2011 [17], Robbins 2010 [47]
Australia, various diseases	2	Marshall 2014 [40], Ward 2010 [52]
Canada, varicella	1	Sweet 2003 [50]
US, seasonal influenza	12	Carpenter 2007 [20], Lott 2012 [37, 38]
US, H1N1	9	Ambrose 2011 [14], Klaiman 2014 [30]
US, hepatitis A and B	4	Mark 2001 [39], Tung 2005 [51]
US, various diseases	2	Limper 2014 [34], Lindley 2008 [35]
UK, HPV	8	Hilton 2011 [25], Potts 2013 [45]
UK, seasonal influenza	1	Kassianos 2015 [29]
UK, hepatitis B	1	Zuckerman 2005 [56]
Worldwide, various diseases	2	Cawley 2010 [21], Cooper Robbins 2011 [23]
TOTAL	44	

We extracted data from the studies and coded findings using the thematic framework of eight organisational factors that we had developed from our scoping review. We compared and contrasted findings within and across studies. One researcher (SP) performed the systematic searches, data extraction, coding and analysis of findings. A sample of ten percent of included and excluded studies was reviewed by a second researcher (ST) to check consistency in the application of the inclusion and exclusion criteria, the quality appraisal criteria, and the coding template. Discrepancies were discussed and agreed between SP and ST.

## Results

### Study selection

**Fig. 1:**
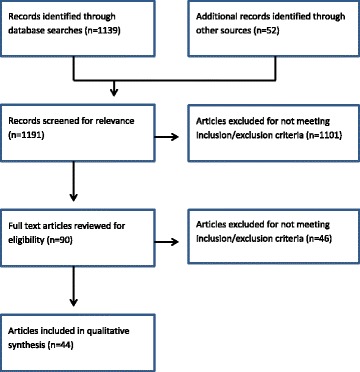
Flow chart for study search and selection process

A total of 44 articles were included in this review [8, 14–56]. The database searches identified 1139 records. A further 52 records were identified from other sources. All 1191 records were screened for relevance by title and abstract (Fig. 1). Of these, 1101 were excluded for not meeting our eligibility criteria, and 90 full text articles were retrieved and reviewed. A final 44 articles met the inclusion criteria and were included in the qualitative synthesis.

### Study characteristics

Of the 44 included studies, the largest proportion (*n* = 16) were descriptive accounts or evaluations of programme experiences, usually written by local or regional programme managers. In addition, there were 15 qualitative studies that used interviews, focus groups, or observations, or a combination of these methods. Seven studies were quantitative, of which three were cohort studies, three used cross-sectional surveys, and one was an economic evaluation. Two studies were systematic reviews, and one an evidence synthesis. The remaining three studies were expert opinion pieces (Table 2). The characteristics of included studies are summarised in a separate file [see Additional file 3].

The majority of studies were conducted in the United States (*n* = 27). There were ten studies from the United Kingdom. The remaining studies were conducted in Australia, Canada or were worldwide reviews (*n* = 7). There were thirteen studies of seasonal influenza, ten of Human Papillomavirus (HPV), and a further nine studies of pandemic influenza (H1N1). Most of the seasonal flu studies (*n* = 12) and all the pandemic flu studies were from the US. Eight of the ten HPV studies were from the UK (Table 3).

We appraised the quality of studies using structured checklists that we adapted from the Critical Appraisal Skills Programme. These checklists are shown in an additional file [see Additional file 2]. They included questions relating to study focus, search strategy, quality assessment of studies, and extraction of results. We found variations in quality and that some studies had a risk of bias. For example, only one third of the qualitative studies had a clear statement of the aims of their research underpinned by a theoretical framework. Only one of the systematic reviews described methods for extracting data and combining results. Only two of the eight quantitative studies adequately considered the potential influence of confounding factors.

### Results of thematic analysis

#### National and regional policy

The first organisational factor that we identified was the impact of national or regional policy on the implementation of programmes. Studies described the influence of two categories of policy — those that had a direct impact on programme delivery, such as the rationale for the programme or the choice of target group, and those that had an indirect and sometimes unanticipated impact on the vaccination programme, such as national policies on education or on health service delivery. Among our studies there was little evaluation of the actual impact policies had; studies tended to describe rather than evaluate the effect of policies on the school vaccination process and programme outcomes.

Several US studies of H1N1 vaccination provided examples of the direct impact that the federal government’s emergency-driven response to pandemic influenza had on the delivery of school-based programmes and public attitudes towards this [14, 28, 30, 32]. For example, one paper explored how the declaration of a public health emergency, and the associated federal legislation that was enacted, gave some schools enough assurance for important programme enablers to be adopted such as school nurses acting as vaccinators [27]. Two papers from the US considered how the public responded to a visible and sometimes prominent government role in vaccination programmes [23, 32]. Cooper Robbins’ systematic review found that the public’s low acceptance of government involvement in immunisation programmes may have decreased uptake [23].

Examples of policies having an indirect influence included a qualitative study from the UK that positioned the school-vaccination programme within a broader context of government policy which favoured schools as a setting for reducing health inequalities and social exclusion. School nurses were expected to play an important role within this broader policy framework [19]. Another descriptive study from the UK briefly described nurses’ fears that recent educational reforms, which allowed for the creation of new schools independent of local government control, might make the organisation of school-based vaccination more difficult [48]. There was already evidence from the UK that school autonomy could indeed be a problem as two faith schools had refused to participate in a school-based HPV programme [8].

#### Programme management and leadership

The second factor — programme management and leadership — was highlighted in several papers. Many studies focused on the influence on programme effectiveness of different types of management and leadership at school or district/area level. We found no studies focusing on national leadership and governance.

Several papers from the US described how the emergency situation created by the pandemic influenza outbreak in 2009 affected the leadership structures of local programmes. In one programme evaluation, H1N1 vaccination planners explained how an incident command process (a standardised hierarchical approach to the management of emergencies) was widely used in the US and was efficient in terms of speed and throughput [27]. Lott described the successful use of a similar model in a non-pandemic flu season where the programme leader was a registered nurse manager familiar with mass clinics for bioterrorism preparedness [37].

In several descriptive studies, programme planners described the importance of school leadership for the effectiveness of programmes. This was a particular theme in the US where securing the full support of the school was important given the power of schools to make decisions about delivering vaccination programmes [26, 38]. This factor was also important in some of the descriptive and qualitative studies from the UK although schools in the UK have little influence over the direction of school-based vaccination programmes [19, 39, 52]. In one such study, school nurses contrasted favourably a school with the highest HPV uptake where the head teacher “dynamically promoted the vaccine” with schools where the vaccination programme was not viewed as a joint responsibility between the health and education service [19, 39, 52]. US studies also identified the importance of local political leadership and endorsement by a senior clinician [46, 53].

The commitment and engagement to the vaccination programme of the school’s entire staff — from administration to teachers to nurses — was a recurring theme in several studies. Hull’s evidence synthesis of seasonal flu vaccination in the US found that having the full support of the school administration and school nurses influenced effectiveness [26]. In a qualitative study of HPV vaccination in Australia, nurses and parents reported that the active involvement of a coordinating teacher influenced parents’ decision to vaccinate [47].

In this example, in common with many other studies in our review, success was not defined which makes interpretation of results difficult. Studies that did define success described it in terms of staff feeling positive, higher vaccine uptake, vaccine series completion or quicker throughput in clinics.

#### Organisational models and institutional relationships

A third factor, related to the previous one on programme leadership, concerned models of programme organisation and inter-organisational relationships. This theme included communication and collaboration across organisations and sectors (for example, between school and health) to achieve the aims of the programme and embedding programmes within local systems.

Some papers from the US identified the strength or weakness of existing organisational relationships as a determinant of programme effectiveness. However, these were descriptive or qualitative studies in which few associations were directly made with success. In 2009 pre-existing partnerships between public health departments and education agencies appeared to allow a rapid response to be mobilised for H1N1 vaccination [27, 30]. Also in the US, school managers and school nurses reported that “good” relationships between the local health department and local school management were necessary for programmes to be effective [30]. Similarly in the UK, where school nurses are usually employed by the health service rather than the school, the quality of the underlying relationship between the school and the school nurse, was important for securing the school’s cooperation with the vaccination programme [19]. Conversely, problems emerged where relationships were weak. One US-based study reported that local doctors were insufficiently engaged in the H1N1 vaccination programme. This led to misconceptions among physicians about the risks of the live influenza vaccine and misleading advice given to parents [20].

Different collaborative models for influenza vaccination were compared in one descriptive study from the US. Instead of adopting a health department-led single model, many schools developed a range of innovations to draw in additional resources. Some models involved collaboration between school nurses across groups of school. Others drew in support (staff or premises) from non-profit and private medical providers and parent volunteers. No single model was associated with a greater likelihood of success. Rather, programmes evolved over consecutive seasons in response to leadership from school staff, and experience of running the programme which built up the confidence for school managers to implement cost-effective improvements [54].

The need for long lead in times — with preparation preferably starting the year before vaccination — was referred to in several descriptive and qualitative papers from the UK and the US, as necessary for developing organisational relationships [22, 26, 38, 54]. Long lead ins allowed for advance visits to schools to take place and early planning to avoid conflicts with school events [27]. However, there were many instances of this not happening in practice. Some UK studies cited examples of new programmes launched by central government and cascaded to local teams and schools with tight timescales. Health professionals, tasked with the introduction of these programmes, voiced concern that programme planning was rushed [29]. This may have led to reduced opportunities for stakeholder engagement.

#### Infrastructure

The fourth factor was infrastructure issues — the facilities and systems needed to operate the programme — with systems for capturing data and for vaccine distribution and supply emerging as the main themes.

Studies from different countries reported on the major organisational challenge arising from school-based programmes not having access to students’ medical records. This was particularly significant for flu campaigns as it meant that school nursing staff could not reliably confirm whether it was safe for children to receive the live attenuated vaccine [27, 32]. Different strategies were deployed to overcome these problems. In some parts of the UK, community nursing staff were contracted to vaccinate students and they had access to students’ clinical records [29]. In Scotland, a centralised child health surveillance system was used to invite children for school vaccinations [45]. In Australia, a state-wide immunisation register was created to facilitate access to individual vaccination records; however, this did not completely resolve the problem as student records were often incomplete [52].

Problems of vaccine supply and local storage were also a recurring theme. In 2009 there were major problems with H1N1 influenza vaccine supply in the US leading to frequent changes in dates and locations of clinics and widespread cancellations of scheduled clinics. In a non-pandemic influenza season, supply issues did not appear to arise. However, studies from the US and the UK reported on the difficulty of reducing vaccine wastage and of managing the cold chain (maintaining vaccines within a correct temperature range from the point of manufacture to use) [29, 37]. Information on the extent to which cold chain errors and vaccine wastage occurred was not however reported in any paper.

#### Workforce: capacity and activity

Workforce was a widely reported factor in studies of different types and from different countries. Recurring themes across countries were staff capacity, workload, skill mix, experience and roles.

Several studies from the UK and the US focused on the important role of the school nurse and his/her attitude to school-based vaccination. Reinforcing the finding on our third theme (organisational models and relationships), strong professional relationships involving the school nurse were often cited as necessary for the development of programmes, and the school nurses’ experience, skills and competence were also key. That experience was useful in the US where school nurses were found to be influential local “connectors” for vaccination programmes, owing to their knowledge of particular students and parents, as well as their ability to educate families about the rationale for vaccination [27]. A similar theme emerged in the UK where school nurses’ familiarity with their students and their established relationships with ‘hard-to reach’ communities were considered important for increasing uptake among girls who did not attend or who missed doses of the vaccine [18].

School nursing capacity was also a recurring theme. Several descriptive and qualitative studies from the US and the UK reported that school nurses felt burdened by the workload associated with school vaccination programmes [19, 25, 39]. One US-based study found that elementary schools whose nurses served more than one school had lower uptake of influenza vaccination than schools with a dedicated nurse. The explanation for this was that nursing time was divided between multiple schools which could affect various aspects of planning and flexibility in clinic scheduling [36]. The same study found that school nursing experience was also important, with school nurses who had more than 20 years’ experience achieving higher uptake. This was thought to be due to the greater knowledge and better relationships that these nurses had with their host schools. Papers also highlighted concerns from school nurses that the vaccination programme took them away from their normal duties including their support for vulnerable pupils, sex education or weight checks [15, 19, 25].

Workforce capacity clearly relates to our next organisational factor on programme financing. Adding to evidence from US papers that many programmes felt under-funded, US studies also reported that it was not possible to run vaccination programmes within existing staff resources. Schools often depended on the use of additional paid staff as well as on volunteers from the Medical Reserve Corps (a network of locally organised health professionals), parents, law enforcement and nursing [26–28, 54].

There were no such ad hoc staffing arrangements in the UK where education and health policies do not easily facilitate volunteer involvement in school health programmes. In some of the UK-based HPV and seasonal influenza programmes, an external immunisation team was recruited to vaccinate in schools. In one study, this model appeared to achieve higher vaccination rates than a school nursing led model. However, its success still depended on the support of the named school nurse for arranging timetables and getting consent forms back, and the external team often felt that this support was not forthcoming [19].

#### Programme financing

The sixth factor — distinct to workforce but overlapping with this theme — was programme financing, and concerned issues of programme funding, billing and reimbursement, and sustainability. This factor featured strongly in the descriptive literature but only from the US which lacks a centrally-funded school-based vaccination programme.

In this context, a number of US-based studies reported the difficulty of securing adequate funding for school-based plans. One study described how local health departments and schools lacked the infrastructure and funding to manage large-scale vaccination programmes. Sometimes private funding was necessary to bridge gaps [46]. The same study highlighted resource inequalities between schools, with some employing full-time school nurses and others sharing school nurses across school districts, and the impact this had on programme delivery — a finding that we also saw in our workforce theme. Managers reported that the H1N1 vaccination campaign would not have been viable without the injection of funds from the federal government and the US Centers for Disease Control (CDC) [27]. A significant barrier to the sustainability of school-based vaccination in the US appeared to be the inability of health departments to bill private insurers and the modest reimbursement rates paid to vaccine providers [34].

#### Communication with parents and students

Most studies, regardless of country or vaccine type, considered communication with parents about the purpose of vaccination and obtaining parental consent as one of the most important factors in the programme’s operation. Many papers in this theme were descriptive and qualitative studies which considered both practical issues, such as distribution of consent forms, and conceptual issues, such as tailoring messages and involving students in shaping programmes. Linking with the capacity theme, the school nurse role in being able to frame and target appropriate messages was influential.

On practical issues, the challenge of consent form distribution and return was a common theme and identified as a significant burden for school administrators. In UK studies of HPV programmes, high rates of consent form return and higher uptake were attributed to the persistent and targeted efforts of school staff and health professionals as well as to repeated opportunities for vaccination [16, 18, 48].

One US study found that using two or more methods to distribute consent forms was effective for improving responses from parents, for example via backpack plus the post [36]. In one programme, leaving too long a gap between dispatch of consent forms and date of vaccination led to confusion and some children being vaccinated twice [22]. However, another study suggested that sending out consent forms at the beginning of the school year, well ahead of the actual vaccination date, encouraged a higher rate of return [37]. Many school sites used consent form templates which were provided by CDC during the H1N1 pandemic [27]. A systematic review and several primary studies from the US found that classroom peer incentives increased consent form return [23, 31, 38]. Elementary schools requiring parental presence and only obtaining consent on the day had lower vaccination rates [36]. Two UK-based studies reported confusion about a young woman’s legal right to make decisions to be vaccinated without parental consent and described this as a barrier to uptake [16, 57].

On more conceptual issues, a repeated theme in the literature was a perception that programme staff needed to tailor messages for different groups of students and parents to avoid inequalities in uptake. For example, the authors of two UK studies of HPV vaccine concluded that more targeted information was needed, as well as flexibility in delivery, in order to respond to HPV vaccination rates that were lower among girls educated in non-mainstream settings. These settings included schools for students with special educational needs and pupil referral units (local government establishments for educating children who cannot attend mainstream schools) [24]. One study found that most questions from parents concerned vaccine side-effects, highlighting the importance of having nurses who were up-to-date in their training and could respond “positively and confidently” [8]. Few US studies picked up this theme. One exception was a descriptive study which suggested that disadvantaged students were more likely to have concerns about vaccine side effects and that this could be a barrier to uptake [20].

Other studies mentioned the challenge of developing age-appropriate materials for older students, although only a few evaluated young people’s views on the information they were given [38, 40, 44]. In fact, children’s and adolescents’ views were largely absent from the literature. Exceptions to this included three studies in the US which found that an important concern for students as well as parents were issues of programme safety and trust in the person giving the vaccine [42, 43, 49].

#### Clinic organisation and delivery

The final factor identified from the literature was the organisation of vaccination clinics. This included on the day logistics and the physical configuration of clinics to facilitate the throughput of students. Preparations for clinics is covered in preceding sections of this paper. Most of the studies that considered clinic organisation were US-based papers on pandemic and seasonal flu.

Among these studies were some that investigated how differences in approaches to clinic organisation influenced outcomes. A few of these, as we have seen, defined a successful outcome as higher uptake or quicker throughput. Different aspects of clinic organisation were understood to contribute towards success defined in these terms. For example, one US-based cohort study found that the principal factors associated with increased uptake in schools of H1N1 vaccination were clinic date (clinics organised earlier rather than later in the season), and clinics conducted during school hours with advance parental consent (rather than after school clinics with parents present) [14].

Other studies that considered clinic organisation were generally descriptive. Good practice was noted but not clearly defined, and the impact of this good practice on a “good” outcome was assumed rather than documented. For example, two US-based studies noted that some schools followed guidelines which set out optimal configurations for clinics to reduce queuing times, such as positioning of work stations and the ratio of support personnel to vaccinators [27, 37]. One of these studies noted that determining the flow and order of classrooms in advance allowed for regulation of the amount of vaccine needing to be thawed in advance [37]. Neither study explained whether greater efficiency was actually achieved by following these practices.

The majority of studies considered outcome in terms of perceptions of success or failure among the staff involved with running the clinics although the personal criteria used by staff for judging a clinic in this way was not clear. In this category of study, local health department staff felt that barriers to the efficient operation of clinics included school nurses not being part of the planning and execution of clinics, as well as problems with vaccine supply and with data collection [27, 28, 30]. Enablers, as has been discussed earlier, included the recruitment of extra staff to assist with the tasks involved in whole-school vaccination [22, 27, 28]. One district in the US had a computer system which assigned clinic volunteers and staff based on clinic distance and areas of expertise [30]. Several school districts used ‘just in time’ training on the day of the vaccination clinic to ensure that volunteers and staff were properly briefed on plans for the day [30].

Reflecting our finding on communication, we found few studies focusing on the clinic experiences of students themselves. The exception was some qualitative research from Australia on the experiences of girls vaccinated against HPV. One such study found that anticipatory fear and distress among girls awaiting vaccination slowed or stopped the process [17, 23]. Some organisational factors were associated with increased fear, for example being vaccinated later in day. Fear could be reduced by methods such as privacy screens, reducing the number of girls waiting for vaccination, vaccinating anxious girls first, the involvement of student peer leaders, and nurses’ use of distraction techniques [23].

## Discussion

We found 44 studies of school-based vaccination that yielded information on the organisational factors that influence the implementation of programmes. Using the thematic framework that we had developed from our initial scoping review, we were able to identify a number of common themes from the literature. Factors that featured strongly in studies included programme leadership and governance, organisational models and institutional relationships, workforce capacity and roles particularly concerning the school nurse, communication with parents and students, including methods for obtaining consent, and clinic organisation and delivery. This is the first time that this information has been brought together and is important for understanding how school-based vaccination programmes work.

These themes relate to each other in a number of ways. For example, funding and staff capacity are clearly linked. In the US, where schools lacked a stable funding stream for seasonal flu vaccination, programmes relied on parent and health service volunteers. In the UK, where there is a centralised funding stream for school programmes, nurses still felt under-resourced and burdened by the additional workload created by vaccination programmes. Managing parental concerns and gaining consent was also a recurring theme, linked closely to workforce capacity and activity, and to inter-organisational relationships. Persistent efforts by committed school and health staff, often working in close collaboration, were needed to maximise high rates of consent form return, high uptake and reduce inequalities.

Our interpretation of the literature is that programmes may work best when all eight organisational factors are positively aligned in a way that facilitates school-based programmes. However, with few rigorous evaluative studies and with studies offering a variety of definitions for ‘successful’ programmes, we cannot be sure that this is the case. Effective programmes appear to be ones with sufficient nursing staff, who are experienced and knowledgeable about immunisation, familiar with the communities with which they work, and able to provide parents with clear confident messages about the safety and benefits of vaccination [18, 27]. Support for nurses to do this appears to come from whole school commitment and involvement starting from school leadership, and backed up by strong professional and institutional relationships between the health and education teams [26]. National policy may either strengthen or weaken the conditions for this to happen. For example, major reform of the education system in the UK, allowing for the creation of state schools independent of local authority control, made the initial implementation of HPV vaccination difficult.

Drawing conclusions is difficult because of the limitations and gaps in the literature. The studies we identified occupy a relatively narrow field dominated by studies of pandemic and non-pandemic influenza vaccination in the US. This limited our ability to consider how different organisational influences affect implementation of programmes in various settings and policy contexts. We recognise that our search strategy may have artificially narrowed the full spectrum of studies published in this area and we discuss this further below. A further constraint we identified is the lack of theory-driven analyses using robust evaluation methods. The preponderance of descriptive papers is problematic. These studies provide detailed accounts of programme experiences, packed with potentially very rich data. However, the quality of these accounts is variable with a high proportion lacking sufficient information about research methods, and a clear definition of outcomes.

A particular feature of the literature is the absence of theories of implementation and organisational change. Exploring this literature would help researchers understand better the organisation and delivery of school-based programmes. For example, academic work on professions and boundaries sheds light on how professional identities are conceptualised and how this can affect intra and inter-professional communication and implementation of new initiatives [58]. This is particularly relevant for our review which found that the role of school nurses and their relationship to other individuals involved in school vaccination featured strongly. Understanding how nurses configure their professional identity and role has implications for school nursing engagement and programme planning. Similarly, theories from improvement research and implementation science can help to explain the enablers and barriers for translation of policy into practice [59, 60]. For school vaccination, these theories can contribute to understanding which factors influence success when national policy on a new immunisation programme is translated into local programmes, and how local planning can seek to maximise the value of these factors. The field of leadership theory, including shared and distributed theories of leadership, is another area which could inform knowledge of effective programmes by helping to explain the influence of different leadership styles on the implementation of school-based vaccination programmes [61]. In one of our studies strong hierarchical leadership appeared to be effective for an emergency pandemic situation [27]. Whether this was effective and acceptable for other scenarios (e.g. more routine delivery) is not clear, as we found that encouraging school engagement is important — suggesting a role for distributed leadership perhaps in combination with aspects of hierarchical approaches.

A further gap we identified was the shortage of studies that considered the views of parents, children and teenagers on the organisational aspects of school programmes, an issue also identified by Cooper Robbins’ systematic review [24]. The few studies that have looked at this issue have found that the way that vaccine clinics are organised can impact on young people’s emotions. Boyce [18, 24] there are strong ethical as well and practical arguments for considering this issue further. The current generation of young people will receive far more vaccines in their lifetime than previous ones. They or their parents may be more or less likely to respond favourably to the next vaccination offered depending on their experience of the school vaccination process.

Some limitations to our study should be considered. Firstly, our searches were confined to studies from high income countries published in English between the years 2000 and 2015. It is likely that this led us to miss relevant studies from other European countries. Limiting our review to 2000–2015 will also have excluded earlier studies of school-based programmes, for example those involving Hepatitis B and TB immunisation. Secondly, our included studies were heterogeneous including formal and informal research and different measures of success. All included studies had been peer-reviewed but quality varied. This variability of methods and limited comparability of findings constrained our ability to draw conclusions from our findings. However, we were interested in the insights that diverse forms of evidence can provide, and we have tried to make the impact of differences in research methods visible by clearly comparing and contrasting results.

## Conclusions

There have been remarkable discoveries in the fields of immunisation and vaccination in recent years, including vaccines for meningitis B, rotavirus, and HPV. Several new vaccines are likely to be added to the childhood immunisation schedule of different countries in the near future [10]. Policy makers will be increasingly drawn to schools as a setting for delivery of these vaccines because of the high vaccination rates that can be achieved. Our study sheds some light on the key organisational influences which impact on the delivery of school-based vaccination.

We identified several organisational factors that are important for the delivery of school-based vaccination programmes, including programme leadership, institutional and professional relationships, and workforce capacity. An understanding of these factors, underpinned by robust theory-informed research, may help policy-makers and managers design and deliver better school-based programmes. We therefore set out the following agenda for future research. Firstly, there is a need for high quality programme evaluations including qualitative studies of processes. Secondly, an increase in studies which are theory-informed, drawing from the literature on theories of implementation and organisational change, will improve understanding of how school-based vaccination programmes work. We have outlined several directions future research might take, such as multi-level studies that explore the interactions between organisational factors (meso), professional roles and identity (micro), and policy imperatives (macro), and how interplay between these levels influences programme implementation. Finally, future research needs to focus on the experiences of children and young people of school-based delivery.

## Additional files

Additional file 1: Example of search strategy used. (DOCX 17 kb)
Additional file 2: Quality checklists used for critical appraisal of included studies. (DOCX 19 kb)
Additional file 3: Characteristics of included studies. (DOCX 37 kb)

## **Abbreviations**

CDC: Centres for disease control (US)
HMIC: Health management information consortium
HPV: Human Papillomavirus
UK: United Kingdom
US: United States (of America)

## References

[1] The complete routine immunisation schedule from summer 2016. London: Public Health England; 2016. https://www.gov.uk/government/publications/the-complete-routine-immunisation-schedule. Accessed 9 Mar 2017.

[2] Paul P, Fabio A (2014). Literature review of HPV vaccine delivery strategies: considerations for school- and non-school based immunization program. Vaccine.

[3] JCVI statement on the annual influenza vaccination programme — extension of the programme to children. UK Joint Committee on Vaccination and Immunisation; 2012. https://www.gov.uk/government/uploads/system/uploads/attachment_data/file/224775/JCVI-statement-on-the-annual-influenza-vaccination-programme-25-July-2012.pdf. Accessed 02 Oct 2015.

[4] Pebody RG (2015). Uptake and impact of vaccinating school age children against influenza during a season with circulation of drifted influenza A and B strains, England, 2014/15. Eurosurveillance.

[5] Pebody RG (2014). Uptake and impact of a new live attenuated influenza vaccine programme in England: early results of a pilot in primary school-age children, 2013/14 influenza season. Eurosurveillance.

[6] Glezen WP (2010). Direct and indirect effectiveness of influenza vaccination delivered to children at school preceding an epidemic caused by 3 new influenza virus variants. J Infect Dis.

[7] Schmier J (2008). Benefits and costs of immunizing children against influenza at school: an economic analysis based on a large-cluster controlled clinical trial. Health Aff (Millwood).

[8] Stretch R (2008). Implementing a school-based HPV vaccination programme. Nurs Times.

[9] Fiala SC (2013). Physician attitudes regarding school-located vaccination clinics. J Sch Health.

[10] Joint Committee on Vaccination and Immunisation. Minutes of meetings held in 2014, 2015 and 2016. https://www.gov.uk/government/groups/joint-committee-on-vaccination-and-immunisation#minutes. Accessed 22 Sept 2016.

[11] Dixon-Woods M (2005). Synthesising qualitative and quantitative evidence: a review of possible methods. J Health Serv Res Policy.

[12] Popay J, Roberts H, Sowden A, Petticrew M, Arai L (2006). Guidance on the conduct of narrative synthesis in systematic reviews: a product from the ESRC methods programme.

[13] Gough D, Thomas J, Oliver S (2012). Clarifying differences between review designs and methods. Syst Rev.

[14] Ambrose CS, Sifakis F (2011). Factors associated with increased vaccination in 2009 H1N1 school-located influenza vaccination programs. Hum Vaccin.

[15] Asay GRB (2012). Coordination costs for school-located influenza vaccination clinics, Maine, 2009 H1N1 pandemic. J Sch Nurs.

[16] Batista Ferrer H, et al. Barriers and facilitators to uptake of the school-based HPV vaccination programme in an ethnically diverse group of young women. J Public Health. 2015. doi:10.1093/pubmed/fdv073. 10.1093/pubmed/fdv073 PMC5072158 26054910

[17] Bernard M (2011). The domino effect: adolescent girls’ response to human papillomavirus vaccination. Med J Aust.

[18] Boyce T, Holmes A (2012). Addressing health inequalities in the delivery of the human papillomavirus vaccination programme: examining the role of the school nurse. PLoS One.

[19] Brabin L (2011). The school nurse, the school and HPV vaccination: a qualitative study of factors affecting HPV vaccine uptake. Vaccine.

[20] Carpenter LR (2007). Mass distribution of free, intranasally administered influenza vaccine in a public school system. Pediatrics.

[21] Cawley J, Hull HF, Rousculp MD (2010). Strategies for implementing school-located influenza vaccination of children: a systematic literature review. J Sch Health.

[22] Christensen JJ (2012). Assessing the acceptability and feasibility of a school-located influenza vaccination program with third-party billing in elementary schools. J Sch Nurs.

[23] Cooper Robbins SC, Ward K, Skinner SR (2011). School-based vaccination: a systematic review of process evaluations. Vaccine.

[24] Fisher H, et al. Examining inequalities in the uptake of the school-based HPV vaccination programme in England: a retrospective cohort study. J Public Health. 2014. doi: 10.1093/pubmed/fdt042. 10.1093/pubmed/fdt042 23620542

[25] Hilton S (2011). School nurses’ experiences of delivering the UK HPV vaccination programme in its first year. BMC Infect Dis.

[26] Hull HF, Ambrose CS (2011). Current experience with school-located influenza vaccination programs in the United States: a review of the medical literature. Hum Vaccin.

[27] Jenlink CH, Kuehnert P, Mazyck D (2010). Key components of a school-located vaccination clinic: lessons learned from fall 2009. J Sch Nurs.

[28] Jenlink CH, Kuehnert P, Mazyck D (2010). Influenza vaccinations, fall 2009: model school-located vaccination clinics. J Sch Nurs.

[29] Kassianos G, et al. Review of the experiences from the first childhood influenza vaccination programme with a live attenuated influenza vaccine in England and Scotland. Drugs Context. 2015;4:212280. 10.7573/dic.212280 PMC4423484 25972905

[30] Klaiman T, O’Connell K, Stoto MA (2014). Learning from successful school-based vaccination clinics during 2009 pH1N1. J Sch Health.

[31] Koff RS (2000). Hepatitis B, school-based vaccination programmes in the USA: a model for hepatitis A and B. Vaccine.

[32] Kuehnert P (2010). Now more than ever: building and sustaining capacity for school-located vaccination initiatives. J Sch Nurs.

[33] Li C, Freedman M, Boyer-Chu L (2009). Championing school-located influenza immunization: the school nurse’s role. J Sch Nurs.

[34] Limper HM (2014). Challenges to school-located vaccination: lessons learned. Pediatrics.

[35] Lindley MC (2008). The role of schools in strengthening delivery of new adolescent vaccinations. Pediatrics.

[36] Lorick SA (2015). Factors associated with uptake of the influenza A(H1N1) pdm09 monovalent pandemic vaccine in K-12 public schools, Maine 2009–2010. J Public Health Manag Pract.

[37] Lott J, Johnson J (2012). Promising practices for school-located vaccination clinics—part II: clinic operations and program sustainability. Pediatrics.

[38] Lott J, Johnson J (2012). Promising practices for school-located vaccination clinics—part I: preparation. Pediatrics.

[39] Mark H, Conklin VG, Wolfe MC (2001). Nurse volunteers in school-based hepatitis B immunization programs. J Sch Nurs.

[40] Marshall HS (2014). Eliciting youth and adult recommendations through citizens’ juries to improve school based adolescent immunisation programs. Vaccine.

[41] Mazyck D (2010). School-located vaccination clinics: then and now. J Sch Nurs.

[42] Middleman AB, Short MB, Doak JS (2012). Focusing on flu: parent perspectives on school-located immunization programs for influenza vaccine. Hum Vaccin Immunother.

[43] Middleman AB, Short MB, Doak JS (2012). School-located influenza immunization programs: factors important to parents and students. Vaccine.

[44] Painter JE (2010). Development, theoretical framework, and lessons learned from implementation of a school-based influenza vaccination intervention. Health Promot Pract.

[45] Potts A (2013). High uptake of HPV immunisation in Scotland — perspectives on maximising uptake. Eurosurveillance.

[46] Ransom J (2009). School-located influenza vaccination clinics: local health department perspectives. J Sch Nurs.

[47] Robbins SC (2010). ‘It’s a logistical nightmare!’ recommendations for optimising human papillomavirus school-based vaccination experience. Sex Health.

[48] Russell M, Raheja V, Jaiyesimi R (2013). Human papillomavirus vaccination in adolescence. Perspect Public Health.

[49] Short MB, Middleman AB (2014). Focusing on flu: adolescents’ perspectives on school-located immunization programs for influenza vaccine. Hum Vaccin Immunother.

[50] Sweet L (2003). Canada’s first universal varicella immunization program: lessons from prince Edward island. Can J Infect Dis.

[51] Tung CS, Middleman AB (2005). An evaluation of school-level factors used in a successful school-based hepatitis B immunization initiative. J Adolesc Health.

[52] Ward KF (2010). School-based vaccination in NSW. N S W Public Health Bull.

[53] Williams V (2012). Elementary school—located influenza vaccine programs: key stakeholder experiences from initiation to continuation. J Sch Nurs.

[54] Wilson D (2013). Implementing and sustaining school—located influenza vaccination programs: perspectives from five diverse school districts. J Sch Nurs.

[55] Wilson T (2001). A bi-state, metropolitan, school—based immunization campaign: lessons from the Kansas city experience. J Pediatr Health Care.

[56] Zuckerman J, Langer B (2005). Hepatitis B vaccination in a school age population: a feasibility study. J Med Virol.

[57] Wood F (2011). What constitutes consent when parents and daughters have different views about having the HPV vaccine: qualitative interviews with stakeholders. J Med Ethics.

[58] Powell A, Davies H (2012). The struggle to improve patient care in the face of professional boundaries. Soc Sci Med.

[59] Øvretveit J (2011). Understanding the conditions for improvement: research to discover which context influences affect improvement success. BMJ Qual Saf.

[60] Greenhalgh T (2004). Diffusion of innovations in service organizations: systematic review and recommendations. Milbank Quarterly.

[61] Turnbull James K (2011). Leadership in context. Lessons from new leadership theory and current leadership development practice. Commission on leadership and management in the NHS.

