# Supplementary material for: School-based vaccination programmes: a systematic review of the evidence on organisation and delivery in high income countries
Source: BMC Public Health. 2017 Mar 14;17:252. doi: 10.1186/s12889-017-4168-0 (PMC5348876; doi:10.1186/s12889-017-4168-0)
Supplement: Additional file 1: — Example of search strategy used. (DOCX 17 kb) [file 12889_2017_4168_MOESM1_ESM.docx]

**Search strategy**

MEDLINE, using the National Institute for Health and Care Excellence ( NICE) Healthcare Databases Advanced Search

1. child*
2. adolescen*
3. school
4. exp MASS VACCINATION
5. vaccination
6. 1 or 2
7. 4 or 5
8. 3 and 6 and 7
9. 8 with limits: Language (English), Publication Date (2000 to 2015)
