# Supplementary material for: School-based vaccination programmes: a systematic review of the evidence on organisation and delivery in high income countries
Source: BMC Public Health. 2017 Mar 14;17:252. doi: 10.1186/s12889-017-4168-0 (PMC5348876; doi:10.1186/s12889-017-4168-0)
Supplement: Additional file 2: — Quality checklists used for critical appraisal of included studies. (DOCX 19 kb) [file 12889_2017_4168_MOESM2_ESM.docx]

**Quality checklists used for critical appraisal of included studies**

We developed three quality checklists using material published by the Critical Appraisal Skills Programme (2014) <http://www.casp-uk.net/> The checklists we used were as follows:

**Checklist for systematic reviews and narrative reviews**

1. Did the review address a clearly focused question?
2. Did the authors look for the right kind of papers?
3. Was sufficient quality assessment of included studies undertaken? Or is the rationale for not undertaking this explained and reasonable?
4. Did the authors explain their methods for extracting data and combining results, and were these methods appropriate?
5. Were results reported clearly?
6. Did the authors explain how their study applies to other settings and groups with reference to previous research/literature?
7. Are the limitations of the study adequately considered?

**Checklist for quantitative studies (used for cohort studies and cross-sectional surveys)**

1. Did the study address a clearly focused question?
2. Was the cohort or sample recruited in ways that were relevant to the research question?
3. Were variables accurately measured to minimise bias?
4. Were all confounding factors taken into account in the design and/or analysis of the study?
5. Were appropriate statistical techniques used to analyse the data?
6. Were the results reported clearly?
7. Did the authors explain how the study applies to other settings and groups with reference to previous research/literature?
8. Are the limitations of the study adequately considered?

**Checklist for qualitative studies**

1. Was there a clear statement of the aims of the research, underpinned by a theoretical framework?
2. Did the authors explain why particular groups or settings were chosen and were reasons given appropriate for the research question?
3. Were methods used to analyse the data explained and were these methods sufficiently rigorous?
4. Did the authors explain how the study applies to other settings and groups with reference to previous research/literature?
5. Are the limitations of the study adequately considered?
