# Supplementary material for: School-based vaccination programmes: a systematic review of the evidence on organisation and delivery in high income countries
Source: BMC Public Health. 2017 Mar 14;17:252. doi: 10.1186/s12889-017-4168-0 (PMC5348876; doi:10.1186/s12889-017-4168-0)
Supplement: Additional file 3: — Characteristics of included studies. (DOCX 37 kb) [file 12889_2017_4168_MOESM3_ESM.docx]

**Characteristics of included studies**

| **Author and year** | **Focus – country, vaccination type, target population, setting** | **Study type** | **Methods** |
| --- | --- | --- | --- |
| Ambrose CS, Sifakis F. (2011) | US  H1N1 influenza virus (2009 pandemic)  All children  Elementary schools | Cohort study | Analysis of government data on factors associated with difference in uptakes between six localities with school-based vaccination programmes. |
| Asay GRB, et al. (2012) | US  H1N1  All children  Elementary and secondary schools | Cohort study | Programme evaluation conducted by the US Centers for Disease Control in nine school districts in Maine to ascertain staff hours and costs associated with "outside-clinic coordination activities". |
| Bernard M, et al. (2011) | Australia  Human Papillomarivus (HPV)  12-16 year old girls  Secondary schools | Qualitative | Qualitative review of experiences of three schools in New South Wales conducted through interviews and focus groups with adolescents, vaccinators and school staff, and observations of clinics. |
| Boyce T, Holmes A. (2012) | UK  HPV  12-13 year old girls  Secondary schools | Qualitative | Rapid evidence review and semi-structured interviews with health professionals in England. |
| Brabin L, et al. (2011) | UK  HPV  12-13 year old girls  Secondary schools | Qualitative | Mixed methods study using cross-sectional survey and interviews with school nurses in two geographical areas. |
| Carpenter LR, et al. (2007) | US  Seasonal influenza  All children  Elementary and secondary schools | Descriptive | Process evaluation of vaccination programmes in schools in Tennessee. |
| Cawley J, Hull HF, Rousculp MD. (2010) | Worldwide, mainly US  All vaccines  All school settings | Review | Systematic review of school-based vaccination programmes worldwide |
| Christensen JJ, et al. (2012) | US  Seasonal influenza  All children  Elementary schools | Qualitative | Structured interviews with school and health staff in New York State concerning the acceptability and feasibility of school-located vaccination for influenza, and experiences of those involved in administering the programme. |
| Cooper Robbins SC, Ward K, Skinner SR. (2011) | Developed countries, mainly US Varicella, HPV, hepatitis B, seasonal influenza, measles, Measles-Mumps-Rubella (MMR)  All school settings | Review | Systematic review of the literature on process evaluations of school-based vaccination programmes |
| Batista Ferrer H, et al. (2015) | UK  HPV  12-13 year old girls  Secondary schools | Qualitative | Observation of school-based vaccination clinics in three schools. Interviews with young women and key informants from the same schools. |
| Fisher H, et al. (2014) | UK  HPV  12-13 year old girls  Secondary schools | Cohort study | Cohort analysis of uptake of vaccine in South West England (14,000 students). |
| Hilton S. (2011) | UK  HPV  12-13 year old girls  Secondary schools | Qualitative | Interviews with school nurses from across the UK. |
| Hull HF, Ambrose CS. (2011) | US  Seasonal influenza  All children  Elementary and secondary schools | Review | Evidence synthesis of published reports of 36 school-based influenza clinics. |
| Jenlink CH, Kuehnert P, Mazyck D. (2010) | US  H1N1  All children  Elementary and secondary schools | Descriptive | Interviews with a selected group of school vaccination planners to review experiences of 2009/10 programmes. |
| Jenlink CH, Kuehnert P, Mazyck D. (2010) | US  H1N1  All children  Elementary and secondary schools | Descriptive | Descriptive account of school-based vaccination programme. |
| Kassianos G, et al. (2015) | UK  Seasonal influenza  All children  Primary and secondary schools | Descriptive | Descriptive account of school-based influenza programme and lessons learned nationwide. |
| Klaiman T, O'Connell K, Stoto MA. (2014) | US  H1N1  All children  Elementary and secondary schools | Qualitative | Comparison of experiences of local health departments with the aim of identifying local health departments that were successful in running school-based clinics. |
| Koff RS. (2000) | US  Hepatitis A and B  All children  Elementary and middle schools | Non-research | Description of three school-based programmes as evidence for feasibility of school-based hepatitis vaccination programmes. |
| Kuehnert P. (2010) | US  H1N1  All children  Elementary and secondary schools | Non-research | Comment from official from county health department. |
| Li C, Freedman M, Boyer-Chu L. (2009) | US  Seasonal influenza  All children  Elementary and secondary schools | Descriptive | Lessons learned and recommendations for the role of the school nurse in influenza vaccination. |
| Limper HM, et al. (2014) | US  All vaccines  Adolescents  Secondary schools | Descriptive | Descriptive accounts of school experiences of creating a financially sustainable school-based system for vaccination of adolescents (HPV, meningococcal conjugate, TdaP – Tetanus Diptheria Pertussis, influenza). |
| Lindley MC, et al. (2008) | US  New vaccines  Adolescents  Secondary schools | Descriptive | Review of role of schools in vaccine programmes, and exploration of their potential to have an expanded role. |
| Lorick SA, et al. (2015) | US  H1N1  All children  Elementary and secondary schools | Cross-sectional survey | Study based on survey responses and using multivariable analyses to examine the association between school and programme factors and H1N1 vaccination rates in schools in Maine. |
| Lott J, Johnson J. (2012a) | US  Seasonal influenza  All children  Elementary and secondary schools | Descriptive | Descriptive account of three years’ experience of school-based influenza programmes in Tennessee (65,000 students). 2012a focuses on preparation. |
| Lott J, Johnson J. (2012b) | US  Seasonal influenza  All children  Elementary and secondary schools | Descriptive | Descriptive account of three years’ experience of school-based influenza programme in Tennessee (65,000 students). 2012b focuses on clinic operations and programme sustainability. |
| Mark H, Conklin VG, Wolfe MC. (2001) | US  Hepatitis B  All children  Middle schools | Descriptive | Descriptive review of school-based hepatitis B vaccination programme in six schools in North Carolina that used volunteer nurses. |
| Marshall HS, et al. (2014) | Australia  All vaccines  Adolescents  Secondary schools | Qualitative | Evaluation of two citizens’ juries (one adult, one youth) convened to consider views on how school-based adolescent vaccination programmes could be improved. |
| Mazyck D. (2010) | US  H1N1  All children  Elementary and secondary schools | Non-research | Comment from official from state health department. |
| Middleman AB, Short MB, Doak JS. (2012) | US  Seasonal influenza  All children  Elementary and secondary schools | Qualitative | Focus groups of parents in six schools in Texas to explore views on school-based programmes. |
| Middleman AB, Short MB, Doak JS. (2012) | US  Seasonal influenza  All children  Secondary schools | Cross-sectional survey | Cross-sectional survey of students and parents in middle and high schools in Texas to explore relative importance of programmatic factors in school-based programmes. |
| Painter JE, et al. (2010) | US  H1N1  All children  Secondary schools | Qualitative | Evaluation of a theory-based educational intervention to increase influenza vaccine uptake through schools in rural Georgia. |
| Potts A, et al. (2013) | Scotland  HPV  All girls  Secondary schools | Descriptive | Description of planning process and implementation of HPV school-based vaccination campaign 2008 – 2010. |
| Ransom J. (2009) | US  Seasonal influenza  All children  Middle and high schools | Descriptive | Descriptive account of the challenges and successes of school-based vaccination clinics. |
| Robbins SC, et al. (2010) | Australia  HPV  12-16 year old girls  Secondary schools | Qualitative | Exploration of participants' experiences of school-based HPV vaccination using semi-structured focus groups with girls, and one-to-one interviews with parents, teachers and nurses. |
| Russell M, Raheja V, Jaiyesimi R. (2013) | UK  HPV  12-13 year old girls  Secondary schools | Descriptive | Descriptive account of parental attitudes, vaccination rates, cost-effectiveness of programmes, and the role of school nurses in delivery of the programme. |
| Short MB, Middleman AB. (2014) | US  Seasonal influenza  All children and adolescents  Middle and high schools | Qualitative | Focus groups in five large schools in Texas. |
| Stretch R. (2008) | UK  HPV  12-13 year old girls  Secondary schools | Qualitative | Review of vaccination programme in two geographical areas, and analysis of parental questions and concerns |
| Sweet L, et al. (2003) | Canada  Varicella  All children  Primary schools | Descriptive | Descriptive account of first universal varicella immunization program in Canada. |
| Tung CS, Middleman AB. (2005) | US  Hepatitis B  All children  Elementary schools | Cross-sectional survey | Cross-sectional survey of school nurses to establish the factors that contribute to the success of school-based programmes in Texas. |
| Ward KF, et al. (2010) | Australia  All vaccines  All children and adolescents  Secondary schools | Descriptive | Historical overview and current analysis of school-based adolescent vaccination programmes in New South Wales. |
| Williams V, et al. (2012) | US  Seasonal influenza  All children  Elementary schools | Qualitative | Qualitative study based on focus groups and structured interviews with teachers, administrators and health care professionals to investigate the logistics and outcomes of influenza programmes in elementary schools. |
| Wilson T. (2001) | US  Hepatitis B  All sixth grade children  Elementary schools | Qualitative | Semi-structured interviews with programme participants in elementary schools in Kansas. |
| Wilson D, et al. (2013) | US  Seasonal influenza  All children  Elementary and secondary schools | Descriptive | Case study methodological approach to describe models for school-based programmes in Los Angeles |
| Zuckerman J, Langer B. (2005) | UK  Hepatitis B  All children  Secondary schools | Cohort study | Prospective cohort study to test feasibility and acceptance of a school-based hepatitis B vaccination programme in two London schools. |

**References**

Ambrose CS, Sifakis F. Factors associated with increased vaccination in 2009 H1N1 school-located influenza vaccination programs. Hum Vaccin. 2011;7:864-7.

Asay GRB, et al. Coordination Costs for School-Located Influenza Vaccination Clinics, Maine, 2009 H1N1 Pandemic. J Sch Nurs. 2012;28:328-335.

Batista Ferrer H, et al. Barriers and facilitators to uptake of the school-based HPV vaccination programme in an ethnically diverse group of young women. Journal of Public Health. 2015; doi: 10.1093/pubmed/fdv073.

Bernard M, et al. The domino effect: adolescent girls' response to human papillomavirus vaccination. The Medical journal of Australia. 2011;194:297-300.

Boyce T, Holmes A. Addressing health inequalities in the delivery of the human papillomavirus vaccination programme: examining the role of the school nurse. PLoS One. 2012;7:9.

Brabin L, et al. The school nurse, the school and HPV vaccination: a qualitative study of factors affecting HPV vaccine uptake. Vaccine. 2011;29:3192-3196.

Carpenter LR, et al. Mass distribution of free, intranasally administered influenza vaccine in a public school system. Pediatrics. 2007;120:172-8.

Cawley J, Hull HF, Rousculp MD. Strategies for implementing school-located influenza vaccination of children: a systematic literature review. J Sch Health. 2010;80:167-75.

Christensen JJ, et al. Assessing the acceptability and feasibility of a school-located influenza vaccination program with third-party billing in elementary schools. J Sch Nurs. 2012;28:344-51.

Cooper Robbins SC, Ward K, Skinner SR. School-based vaccination: a systematic review of process evaluations. Vaccine. 2011;29:9588-99.

Fisher H, et al. Examining inequalities in the uptake of the school-based HPV vaccination programme in England: a retrospective cohort study. Journal of public health. 2014; doi: 10.1093/pubmed/fdt042.

Hilton S. School nurses' experiences of delivering the UK HPV vaccination programme in its first year. BMC Infectious Diseases. 2011;11:226.

Hull HF, Ambrose CS. Current experience with school-located influenza vaccination programs in the United States: a review of the medical literature. Hum Vaccin. 2011;7:153-60.

Jenlink CH, Kuehnert P, Mazyck D. Key components of a school-located vaccination clinic: lessons learned from fall 2009. J Sch Nurs. 2010;26 Suppl 4:14-26.

Jenlink CH, Kuehnert P, Mazyck D. Influenza Vaccinations, Fall 2009: Model School-Located Vaccination Clinics. J Sch Nurs. 2010;26 Suppl 4:7-13.

Kassianos G, et al. Review of the experiences from the first childhood influenza vaccination programme with a live attenuated influenza vaccine in England and Scotland. Drugs in context. 2015;4.

Klaiman T, O'Connell K, Stoto MA. Learning from successful school-based vaccination clinics during 2009 pH1N1. J Sch Health. 2014;84:63-9.

Koff RS. Hepatitis B school-based vaccination programmes in the USA: a model for hepatitis A and B. Vaccine. 2000;18 Suppl 1:77-9.

Kuehnert P. Now more than ever: building and sustaining capacity for school-located vaccination initiatives. J Sch Nurs. 2010;26 Suppl 4:27-29.

Li C, Freedman M, Boyer-Chu L. Championing school-located influenza immunization: the school nurse’s role. J Sch Nurs. 2009;25 Suppl 1:18-28.

Limper HM, et al. Challenges to school-located vaccination: lessons learned. Pediatrics. 2014; 134:803-8.

Lindley MC, et al. The role of schools in strengthening delivery of new adolescent vaccinations. Pediatrics. 2008;121 Suppl 1:46-54.

Lorick SA, et al. Factors associated with uptake of the influenza A(H1N1) pdm09 monovalent pandemic vaccine in K-12 public schools, Maine 2009-2010. Journal of Public Health Management and Practice. 2015;21:186-195.

Lott J, Johnson J. Promising practices for school-located vaccination clinics--part I: preparation. Pediatrics. 2012;129 Suppl 2:75-80.

Lott J, Johnson J. Promising practices for school-located vaccination clinics-- part II: clinic operations and program sustainability. Pediatrics. 2012;129 Suppl 2:81-7.

Mark H, Conklin VG, Wolfe MC. Nurse volunteers in school-based hepatitis B immunization programs. J Sch Nurs. 2001;17:185-188.

Marshall HS, et al. Eliciting youth and adult recommendations through citizens' juries to improve school based adolescent immunisation programs. Vaccine. 2014;32:2434-40.

Mazyck D. School-located vaccination clinics: then and now. J Sch Nurs. 2010;26 Suppl 4:3-6.

Middleman AB, Short MB, Doak JS. Focusing on flu: parent perspectives on school-located immunization programs for influenza vaccine. Hum Vaccin Immunother. 2012;8:1395-400.

Middleman AB, Short MB, Doak JS. School-located influenza immunization programs: factors important to parents and students. Vaccine. 2012;30:4993-4999.

Painter JE, et al. Development, theoretical framework, and lessons learned from implementation of a school-based influenza vaccination intervention. Health Promot Pract. 2010;11 Suppl 3:42-52.

Potts A, et al. High uptake of HPV immunisation in Scotland - perspectives on maximising uptake. Eurosurveillance. 2013;18:39.

Ransom J. School-located influenza vaccination clinics: local health department perspectives. J Sch Nurs. 2009;25 Suppl 1:13-17.

Robbins SC, et al. 'It's a logistical nightmare!' Recommendations for optimising human papillomavirus school-based vaccination experience. Sex Health. 2010;7:271-8.

Russell M, Raheja V, Jaiyesimi R. Human papillomavirus vaccination in adolescence. Perspectives in Public Health. 2013;133:320.

Short MB, Middleman AB. Focusing on flu: adolescents' perspectives on school-located immunization programs for influenza vaccine. Hum Vaccin Immunother. 2014;10:216-23.

Stretch R. Implementing a school-based HPV vaccination programme. Nurs Times. 2008;104: 30-33.

Sweet L, et al. Canada's first universal varicella immunization program: lessons from Prince Edward Island. Can J Infect Dis. 2003;14:41-4.

Tung CS, Middleman AB. An evaluation of school-level factors used in a successful school-based hepatitis B immunization initiative. J Adolesc Health. 2005;37:61-8.

Ward KF, et al. School-based vaccination in NSW. N S W Public Health Bull. 2010;21:237-42.

Williams V, et al. Elementary school–located influenza vaccine programs: key stakeholder experiences from initiation to continuation. J Sch Nurs. 2012;28:256-267.

Wilson D, et al. Implementing and sustaining school-located influenza vaccination programs: perspectives from five diverse school districts. J Sch Nurs. 2013;29:303-314.

Wilson T. A bi-state, metropolitan, school-based immunization campaign: lessons from the Kansas City experience. J Pediatr Health Care. 2001;15:173-8.

Zuckerman J, Langer B. Hepatitis B vaccination in a school age population: a feasibility study. Journal of medical virology.2005;76:47.
